# Supplementary material for: The Cultural and Commercial Value of Tulsi (Ocimum tenuiflorum L.): Multidisciplinary Approaches Focusing on Species Authentication
Source: Plants (Basel). 2022 Nov 18;11(22):3160. doi: 10.3390/plants11223160 (PMC9692689; doi:10.3390/plants11223160)
Supplement: Supplementary file 1 [file plants-11-03160-s001.zip › plants-1817464-supplementary.pdf]

plants

MDPI

Supplementary material S1 – creating a contig

Section of a contig produced from three electropherograms of a reference *O. tenuiflorum* sample.
The electropherograms of three ITS sequences (one reverse ITS 4 and two forward ITS 1 reads) for reference sample G26.

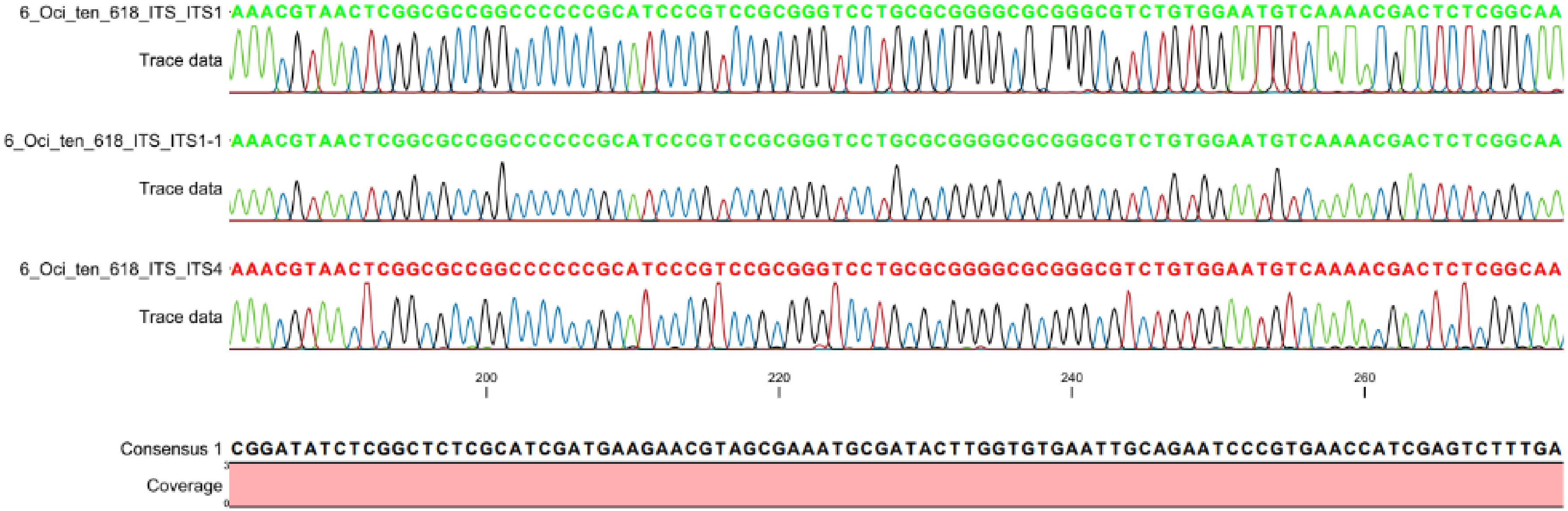

Supplementary material S2 – Multiple alignment of reference ITS sequences from reference sources and GenBank.

A multiple alignment showing different Ocimum samples aligned to illustrate similarities and differences in sequences obtained and from reference sources and Genbank.

Key: letter of sample source*_reference number_barcode region

*G = Professor Peter Nick (Botanical Institute Karlsruhe Institute of Technology, Germany); V = Dr. Eike Reich (CAMAG Laboratory, Switzerland); C = The Royal Botanic Gardens Kew – DNA bank.

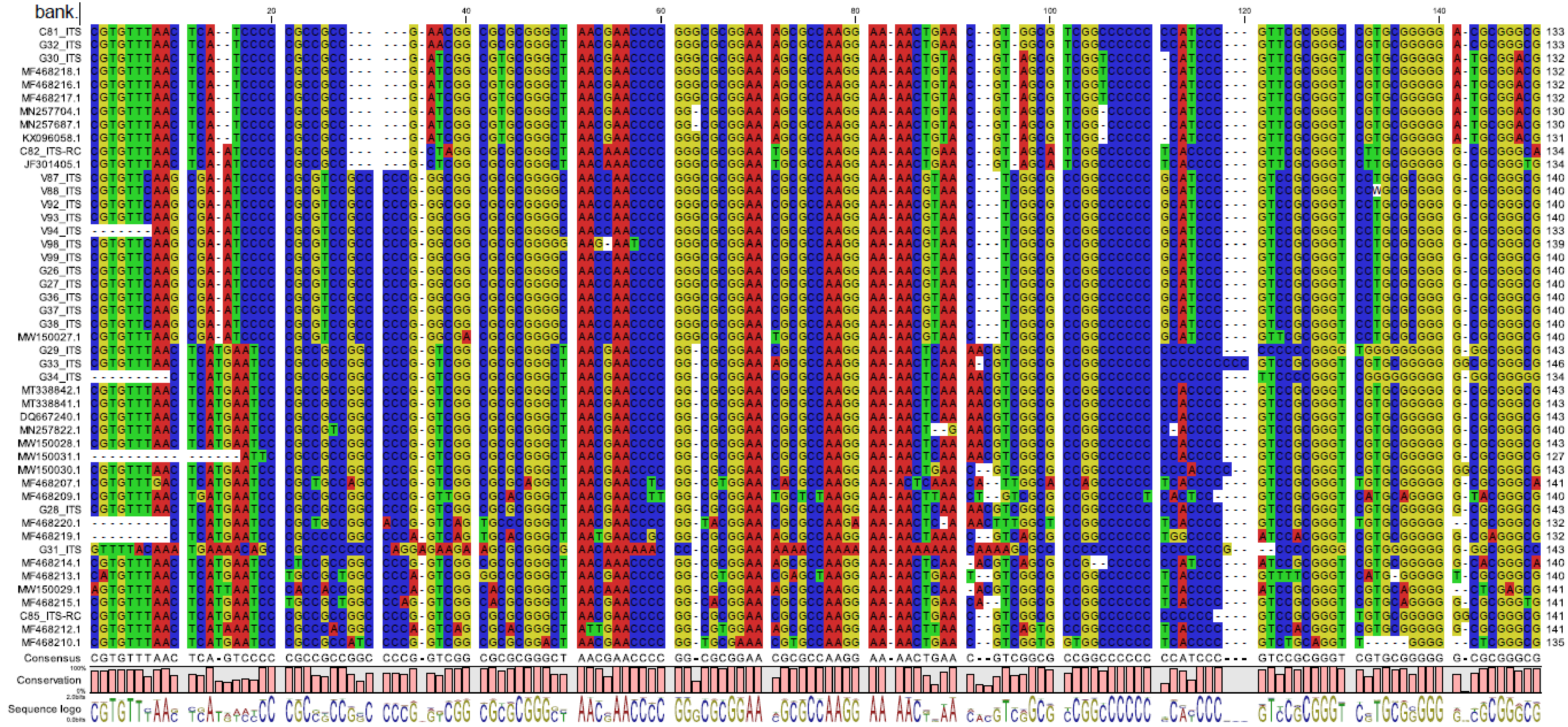

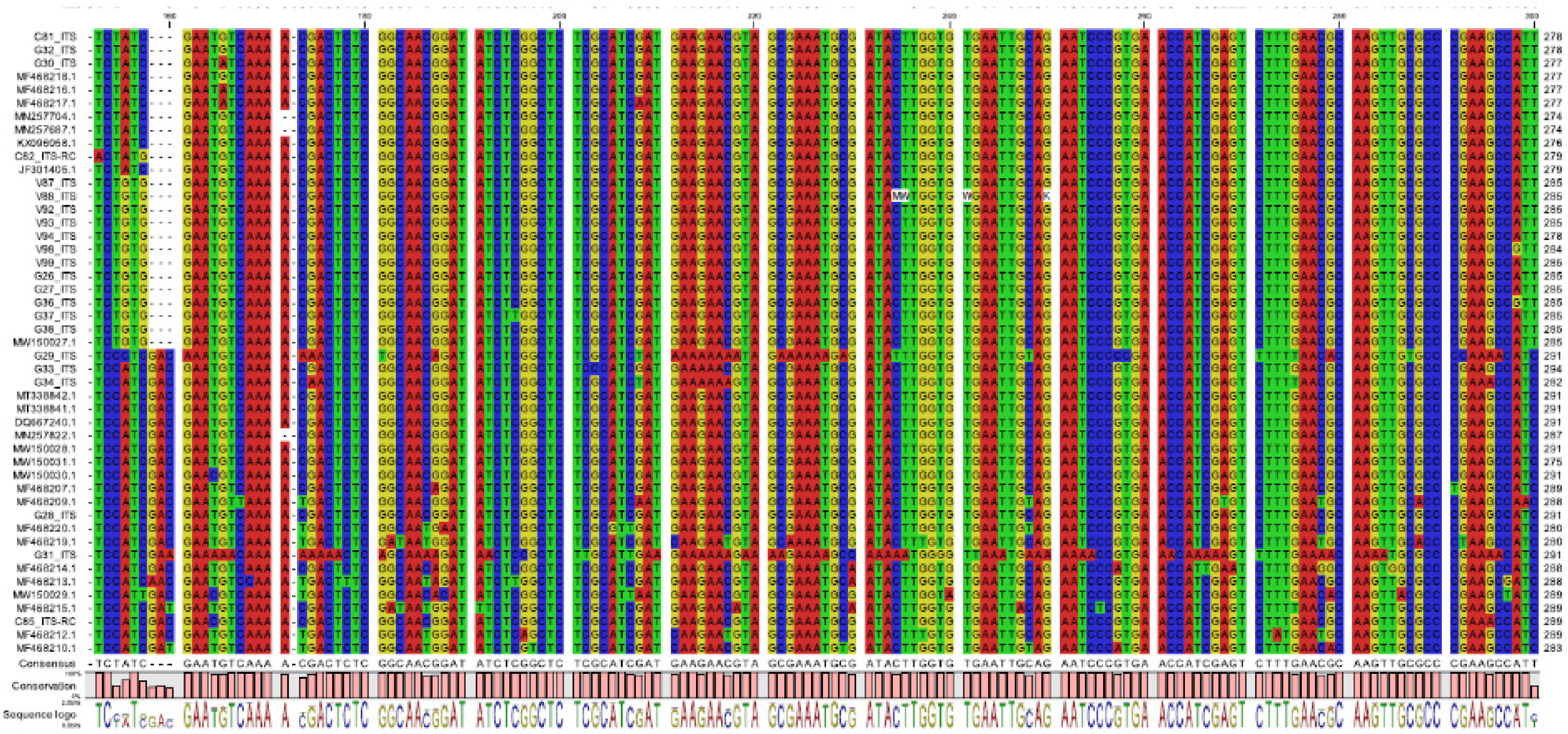
Consensus
Conservation
Sequence logo

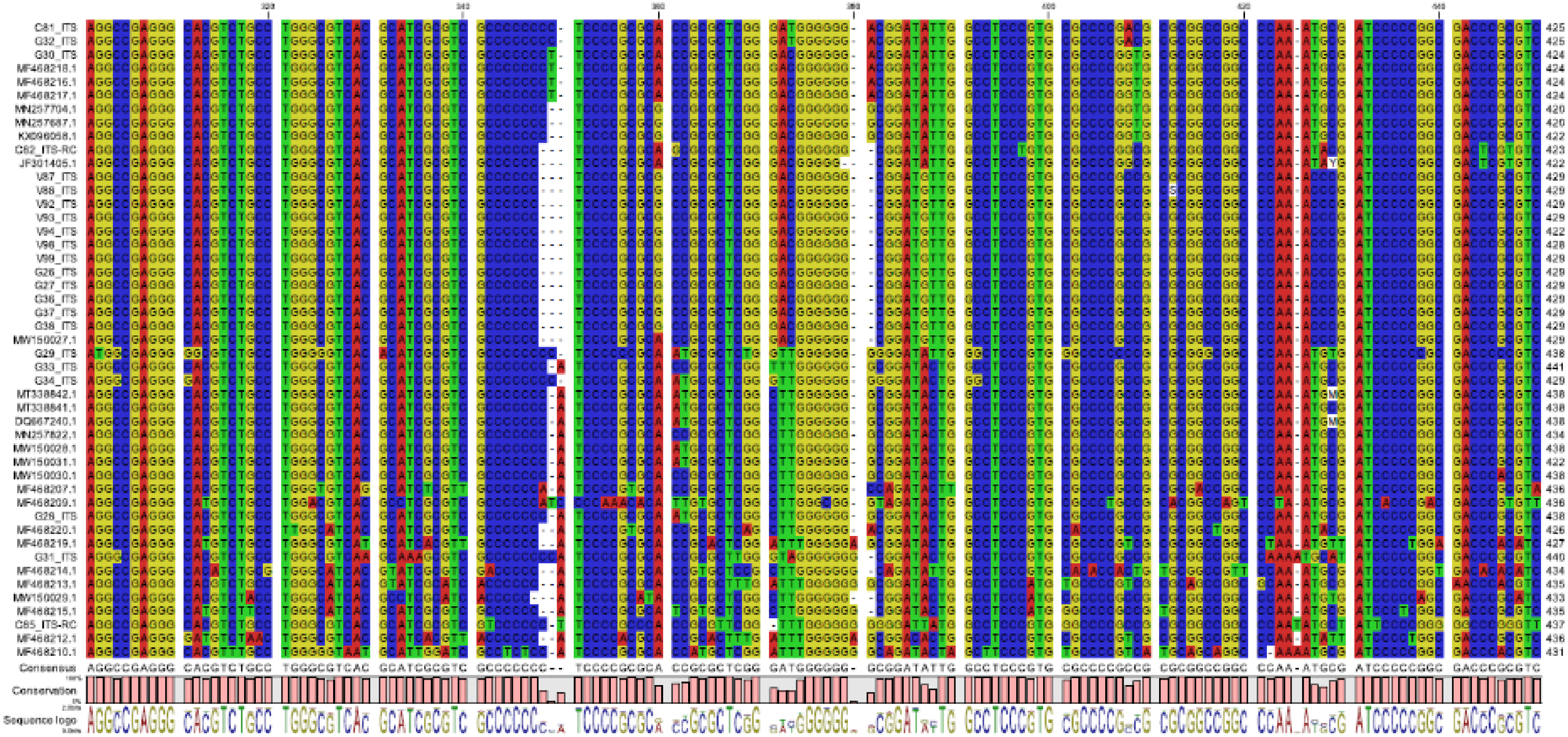
Consensus
Conservation
Sequence logo

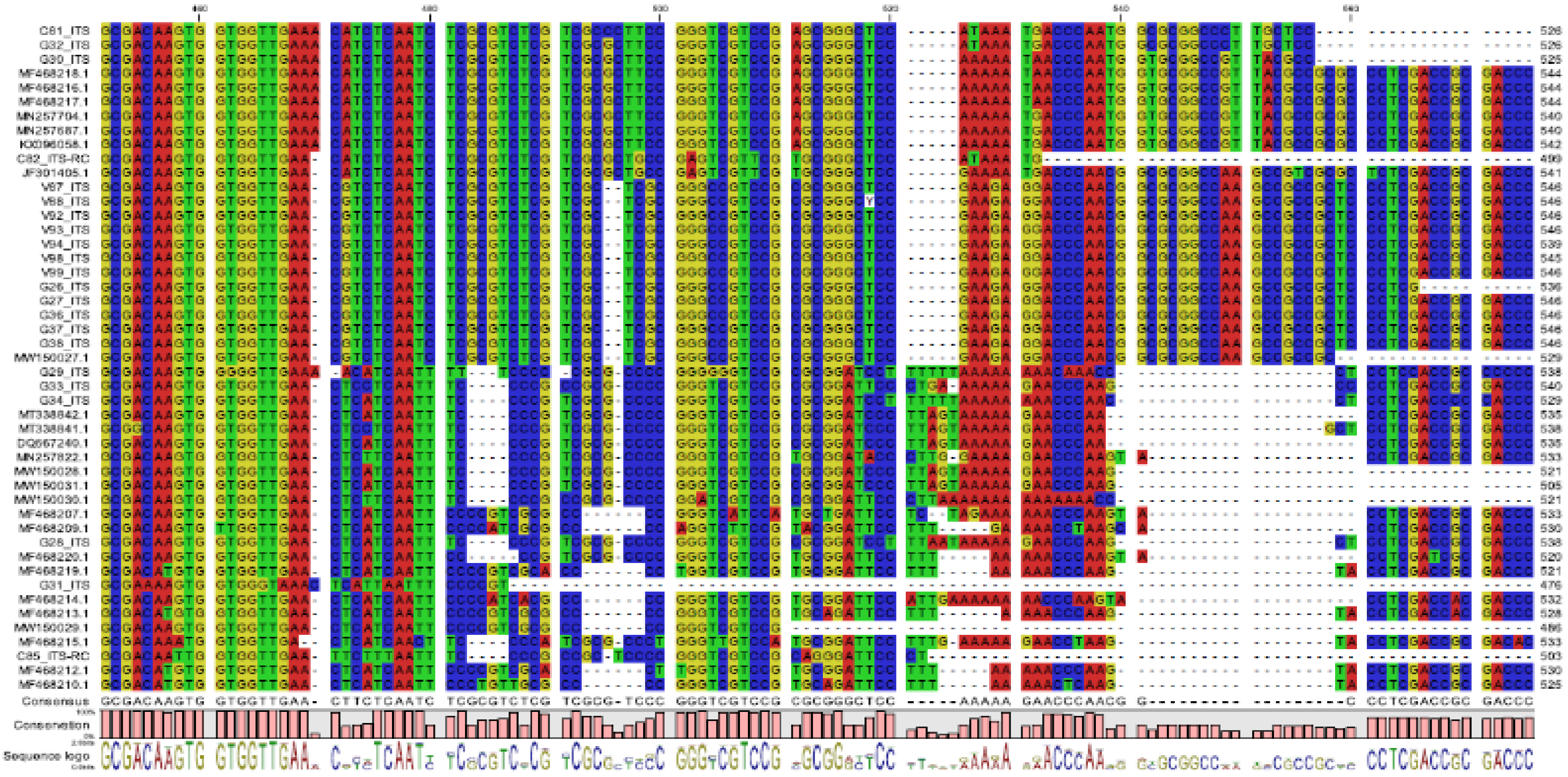
Consensus
Conservation
Sequence logo

plants

MDPI

Supplementary material S3 – Multiple alignment of community Raam and Shyam Tulsi samples aligned with reference samples.

A section of a multiple alignment of community Raam and Shyam Tulsi samples aligned with reference *O. tenuiflorum* and *O. gratissimum* sequences (ITS region). The alignment highlights some of the key differences between *O. tenuiflorum* and *O. gratissimum* and how the two different community samples align with the reference sequences.

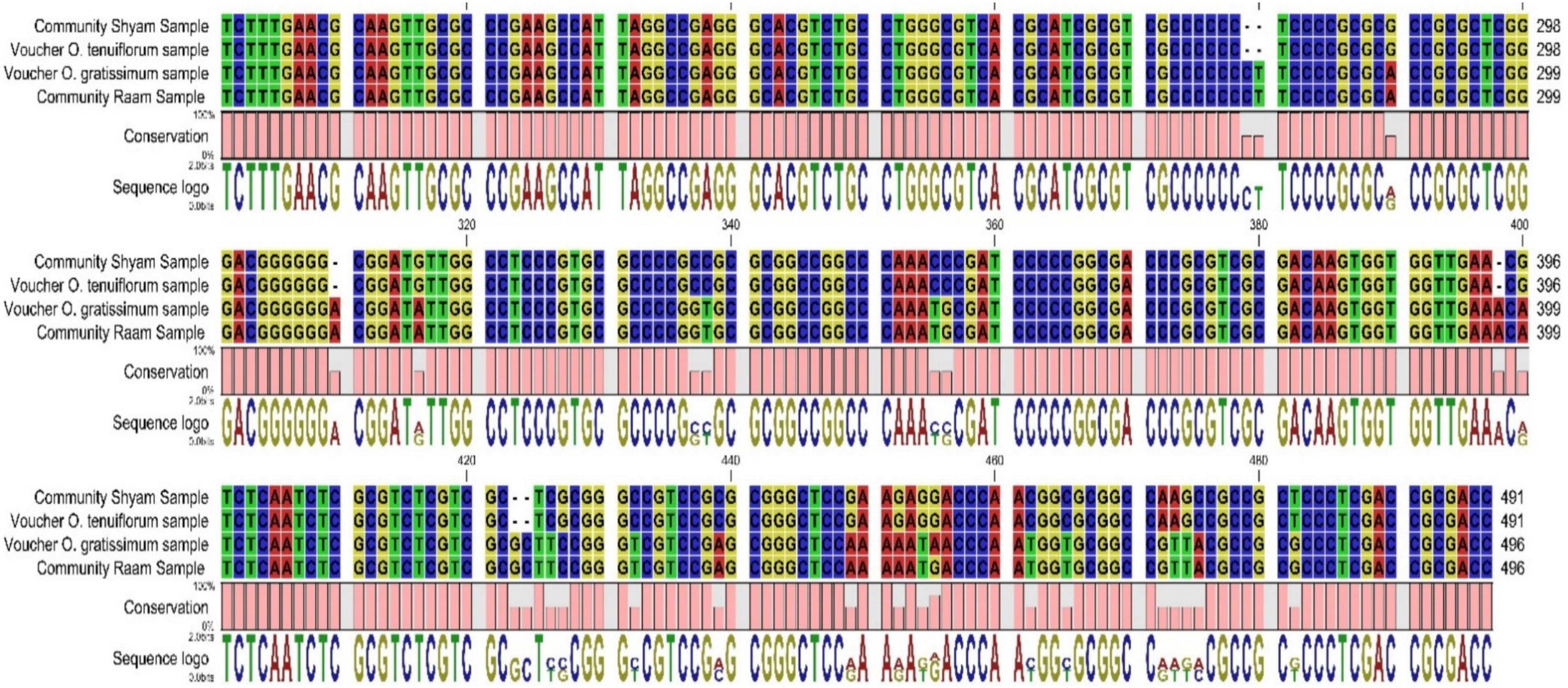

Supplementary material S4 – Multiple alignment of a reference O. tenuiflorum ITS sequence aligned with several unidentified Tulsi ample sequences

Section of a multiple alignment of a reference *O. tenuiflorum* ITS sequence aligned with several unidentified Tulsi sample sequences.
The multiple alignment represents a reference *O. tenuiflorum* sequence (a consensus of seven different reference samples) aligned with several Tulsi samples for which the species were unknown (Oci_unk). Key: *Oci_unk_ sample identification code_extraction number_ region amplified*

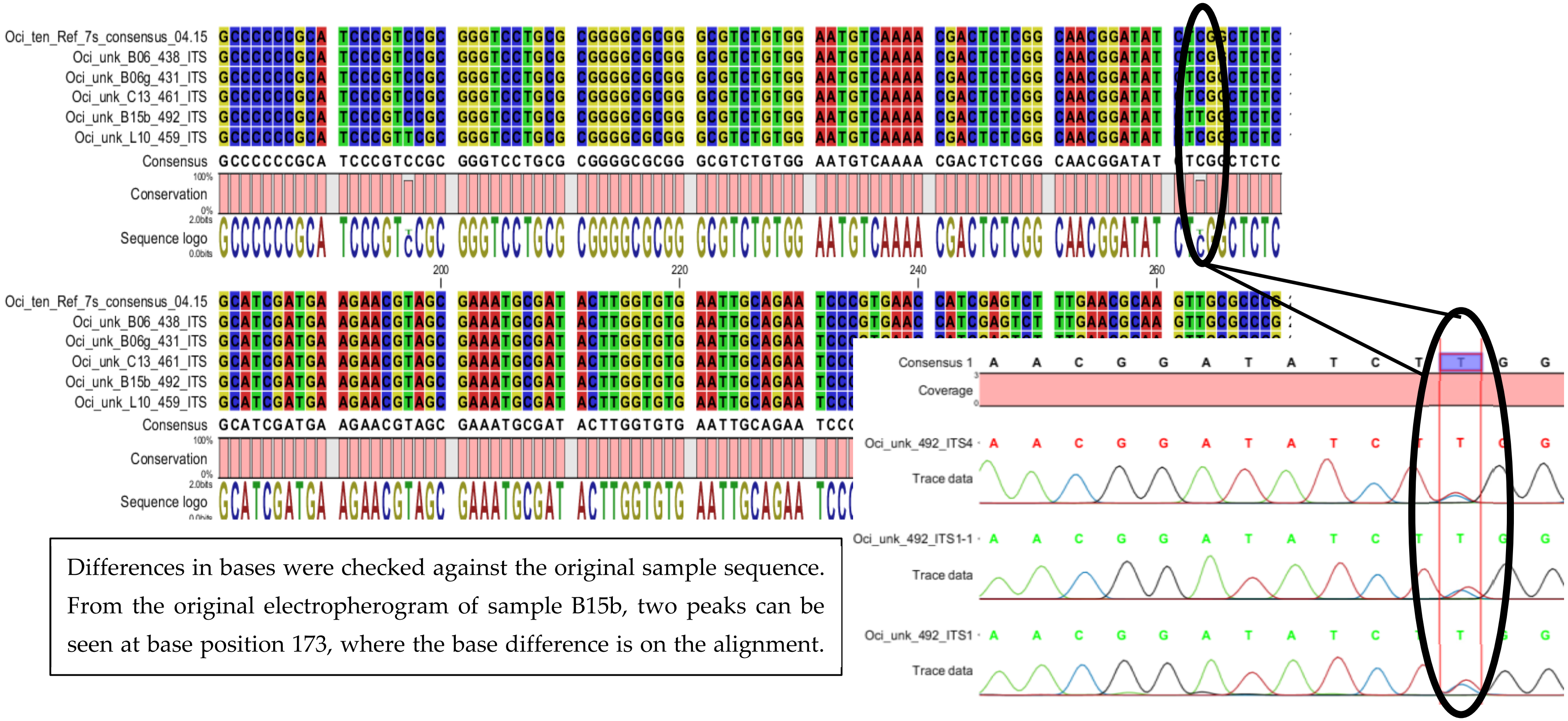

Differences in bases were checked against the original sample sequence. From the original electropherogram of sample B15b, two peaks can be seen at base position 173, where the base difference is on the alignment.

Supplementary material S5 – Reference *Ocimum* sequences, nuclear internal transcribed spacer (ITS1 and ITS2)

All DNA extractions were conducted on fresh leaf samples, grown at De Montfort University. Seeds were obtained from the Botanical Institute Karlsruhe Institute of Technology, Germany. Sequences are consensus of three independent reads in the forward and reverse directions.

Species	Sample ID	DNA Sequence	Bp	Image
Ocimum africanum	G28	CGTGTTTAACTCATGAATCCCGCCGCCGGCCCCGGTCGGCGCGCGGGCTAA<br>CGAACCCCGGCGCGGAACGCGCCAAGGAAAACTCAAAACGTCGGCGCCG<br>GCCCCCCCCACCCCGTCCGCGGGTCGTGCGGGGGGCGCGGGCGTCCATCGA<br>CGAATGTCAAAACGACTCTCGGCAACGGATATCTCGGCTCTCGCATCGATG<br>AAGAACGTAGCGAAATGCGATACTTGGTGTGAATTGCAGAATCCCGTGAAC<br>CATCGAGTCTTTGAACGCAAGTTGCGCCCGAAGCCATCAGGCCGAGGGCAC<br>GTCTGCCTGGGCGTCACGCATCGCGTCGCCCCCCCATCCCCGCGCAATGCG<br>CTCGGCTTGGGGGGGCGGATACTGGCCTCCCGTGCGCCCCGCCGCGCGGCC<br>GGCCCAAATGCGATCCCCCGGCGACCCGCGTCGCGACAAGTGGTGGTTGA<br>ACTCATCAATTTCCCCGTCGCGCCCCGGGTCGTCCGCGCGGATCCTTTAATA<br>AAAAGAACCCAAGCTCCTCGACCGCGACC	537	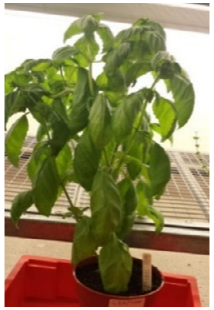
Ocimum basilicum	G34	CTCATGAATCCCGCCGCCGGCCCCGGTCGGCGCGCGGGCTAACGAACCCCG<br>GCGCGGAACGCGCCAAGGAAAACTCAAAACGTCGGCGCCGGCCCCCCCCC<br>CCCCTTCCCCGGGTCGGGGGGGGGGGCGGGGGTCCATCGACGAATGTCA<br>AAACAACTCTCGGCAACGGATATCTCGGCTCTCGCATCTATGAAAAAAGTA<br>GCGAAATGCGATACTTGGTGTGAATTGCAGAATCCCGTGAACCATCGAGTC<br>TTTTAACGCAAGTTGCGCCCGAAACCATCAGGGCGAGGGGACGTCTGCCTG<br>GGCGTCACGCATCGCGTCGCCCCCCCCTCCCCGCGCAATGCGCTCGGGTTG<br>GGGGGGGGGATACTGGGCTCCCGTGCGCCCCGCCGCGCGGCCGGCCCAAA<br>TGCGATCCCCCGGCGACCCGCGTCGCGACAAGTGGTGGTTGAACTCATCAA<br>TTTCCCCGTCGCGCCCCGGGTCGTCCGCGCGGATCCTTTTTTAAAAAAAACC<br>CAACCTCCTCGACCCCCACC	528	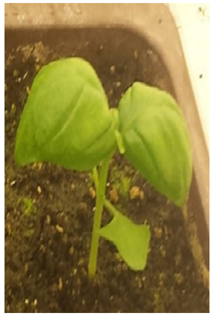

Ocimum citridorium	G29	CGTGTTTAACTCATGAATCCCGCCGCCGGCCCCGGTCGGCGCGCGGGCTAA<br>CGAACCCCGGCGCGGAACGCGCCAAGGAAAACTCAAAACGTCGGCGCCG<br>GCCCCCCCCCCCCCCCCCCCGGGGTGGGGGGGGGGGCGGGCGTCCCTCG<br>ACAAATGTCAAAAAAACTCTCTGCAACAGATATCTCGGCTCTCGCATCTAT<br>AAAAAAAATAGAAAAAAGAGATATTTGGTGTGAATTGTAGAATCCCCCGA<br>ACCATCGAGTTTTTTAACACAAGTTGTGCCCCAAAACATCATGGCGAGGGG<br>GCGTCTGCCTGGGGGTCACACATCGCGTCGCCCCCCCCTCCCCCCGCAATG<br>CGCTCTGGTTGGGGGGGGGGATATTGGGCTCCCGTGGGCCCCCCCGCGCGG<br>GCGGCCCAAATGTGATCCCCCCGCGACCCCCGTCGCGACAAGTGGGGGTTG<br>AAAACATCAATTTTTCCCCCGCGCCCCGGGGGGTCCGCGCGGATCCTTTTTT<br>AAAAAAAACAAACCCTCCTCCACCGCCCCCC	538	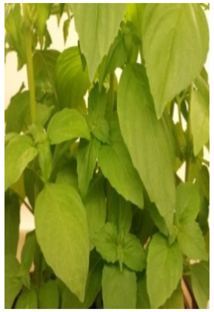
Ocimum gratissimum	G30	CGTGTTTAACTCATCCCCCGCCGCCGATCGGCGTGCGGGCTAACGAACCCC<br>GGGCGCGGAAAGCGCCAAGGAAAACTGTACGTAGCGTCGGTCCCCCCATC<br>CCGTTCGCGGGTCGTGCGGGGGATGCGGACGTCTATCGAATATCAAAACGA<br>CTCTCGGCAACGGATATCTCGGCTCTCGCATCGATGAAGAACGTAGCGAAA<br>TGCGATACTTGGTGTGAATTGCAGAATCCCGTGAACCATCGAGTCTTTGAAC<br>GCAAGTTGCGCCCGAAGCCATTAGGCCGAGGGCACGTCTGCCTGGGCGTCA<br>CGCATCGCGTCGCCCCCCCTTCCCCGCGCACCGCGCTCGGGACGGGGGAC<br>GGATATTGGCCTCCCGTGCGCCCCGGTGCGCGGCCGGCCCAAATGCGATCC<br>CCCGGCGACCCGCGTCGCGACAAGTGGTGGTTGAAACATCTCAATCTCGCG<br>TCTCGTCGCGCTTCCGGGTCGTCCGAGCGGGCTCCAAAAATAACCCAATGG<br>TGCGGCCGTTACGCC	525	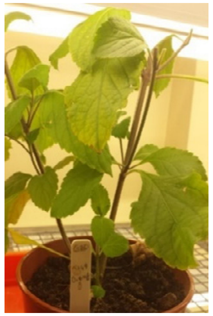

Ocimum kilimandscharicum	G31	GTTTTACAAATGAAAACAGCCGCCCCCCCCCAGGAGAAGAAGCGCGGGCG<br>AACAAAAAACCCCGCGGAAAAAACCAAAAAAAAAAAAAACAAAAGCGCC<br>CCCCCCCCCCCCCCCCGCCCCGGGGCGTGGGGGGGGGCGGCCTCCATC<br>GAAGAAAAACAAAAAAAAACTCAGCAAAAGATAACTCCGCTCTTGCATTG<br>AAGAAAAAAGAAAAGAAAAGCCAAAAATGGGGTTAAATGAAAAAAACCG<br>TGAAACAAAAAGTTTTTGAAAACAAAATGCGCCCGAAAACATCAGGGCGA<br>GGGCACGTCTGCCTGGGCGTCAAGCAAAGCGTCGCCCCCCCCATCCCCGCG<br>CACCGCGCTTGGGTAGGGGGGGCGGATACTGGCCTCCCGTGCGCCCCGCCG<br>CGCGGCCGGCCAAAATGCATATCCCCCGGCGACCCGCGTCGCGAAAAGTG<br>GTGGGTAAACTCATTAATTTCCCCGT	476	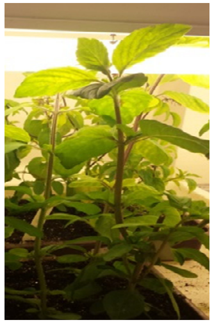
Ocimum tenuiflorum	G26	CGTGTTCAAGCGAATCCCCCGCGTCCGCCCCCGGGCGGCGCGCGGGGCAA<br>CCAACCCCGGGCGCGGAACGCGCCAAGGAAAACGTAACTCGGCGCCGGCC<br>CCCCGCATCCCGTCCGCGGGTCCTGCGCGGGGCGCGGGCGTCTGTGGAATG<br>TCAAAACGACTCTCGGCAACGGATATCTCGGCTCTCGCATCGATGAAGAAC<br>GTAGCGAAATGCGATACTTGGTGTGAATTGCAGAATCCCGTGAACCATCGA<br>GTCTTTGAACGCAAGTTGCGCCCGAAGCCATTAGGCCGAGGGCACGTCTGC<br>CTGGGCGTCACGCATCGCGTCGCCCCCCTCCCCGCGCGCCGCGCTCGGGAC<br>GGGGGGCGGATGTTGGCCTCCCGTGCGCCCCGCCGCGCGGCCGGCCCAAA<br>CCCGATCCCCCGGCGACCCGCGTCGCGACAAGTGGTGGTTGAACGTCTCAA<br>TCTCGCGTCTCGTCGCTCGCGGGCCGTCCGCGCGGGCTCCGAAGAGGACCC<br>AACGGCGCGGCCAAGCCGCCGCTCCCTCGACCGCGACCC	546	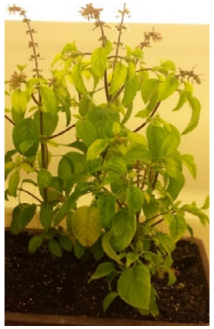

*Images by S.K.Bhamra
